# Randomized Controlled Clinical Study to Determine the Oral and Dermal Tolerability of an Experimental Denture Wipe

**DOI:** 10.1111/jopr.12992

**Published:** 2018-11-28

**Authors:** Alyson Axe, Gary R. Burnett, Kimberly R. Milleman, Avinash Patil, Jeffery L. Milleman

**Affiliations:** ^1^ GSK Consumer Healthcare Weybridge Surrey UK; ^2^ Salus Research Inc. Fort Wayne IN; ^3^ Syneos Health Pune India

**Keywords:** Denture hygiene, safety

## Abstract

**Purpose:**

To evaluate oral and dermal tolerance following use and user acceptability of an experimental denture‐cleansing wipe. An exploratory objective was to develop a method to assess denture wipe effectiveness in removing debris from denture surfaces.

**Materials and Methods:**

This was a single‐center, randomized, controlled, parallel‐group, examiner‐blind study in participants with ≥1 full/partial denture. Participants were randomized to clean their dentures with the denture wipe (n = 76) or water (n = 76) up to 4 times per day for 14 days. Tolerability was assessed by treatment‐emergent adverse events (TEAEs), oral soft tissue examination, and lead hand dermatological assessment. Acceptability was assessed by questionnaire. The feasibility of a methodology to assess the efficacy of the wipe at removing food particles was also evaluated through determination of the mass of chewed peanut particles that the wipe removed after a single use (n = 31).

**Results:**

The proportion of participants experiencing oral TEAEs by day 14 was 0.039% with the denture wipe (lip injury [n = 1], mouth injury [n = 2]) and 0.013% with the water rinse (coated tongue [n = 1]). There were no dermal TEAEs and no TEAE‐related study withdrawals. Skin irritation scores with the denture wipe remained unchanged from baseline. Comparing before vs. after cleaning with the denture wipe, a higher proportion of participants rated their dentures as feeling extremely/very fresh (28.9% pre‐/85.5% post‐cleaning), feeling extremely/very clean (34.2%/86.8%) and looking extremely/very clean (43.5%/85.5%). More denture‐wipe group participants than water‐rinse group participants were extremely/very satisfied with the amount of debris removed from their dentures (88.1% vs 72.4%). The methodology used to assess the weight of peanut particles captured from the wipes/dentures appeared to be a feasible investigation technique.

**Conclusions:**

The denture wipe was generally well‐tolerated and had good user acceptability. The methodology for assessing the mass of peanut particles removed by denture wipes was successful.

Poor denture hygiene is a common problem for denture wearers and can negatively impact both oral and general health, causing gum disease, halitosis, and local and systemic infection.^1^, ^2^, ^3^, ^4^, ^5^ During mastication, food particles can accumulate underneath and on the surface of the denture and, without appropriate denture cleaning, can lead to the formation of a denture plaque/biofilm.^4^, ^6^ The denture biofilm is a reservoir of accumulating complex combinations of opportunistic microorganisms that can lead to the development of local infections, most commonly *Candida*‐related stomatitis.^4^ Furthermore, poor denture hygiene has been associated with denture colonization by oral microorganisms involved in the development of systemic diseases, including bacterial endocarditis and aspiration pneumonia.^3^, ^7^, ^8^, ^9^, ^10^ Effective denture and oral hygiene therefore plays an important role in preventing disease in denture wearers.

Common denture cleaning practices include rinsing with tap water, brushing with toothpaste, and soaking dentures in cleansing tablets dissolved in water.^11^, ^12^ When away from home, the main methods employed are rinsing under a tap or cleaning dentures with a brush and toothpaste. Although effective in maintaining denture cleanliness, these methods may be difficult to implement when away from the home due to the requirement for a source of water. Sterile, disposable denture wipes could therefore offer denture wearers a convenient and time‐efficient method of denture cleaning when they are unable to complete a total conventional cleaning and would also allow wearers the opportunity to easily refresh their dentures at regular intervals throughout the day.

An experimental denture wipe has been developed comprising a nonwoven viscose/polypropylene material impregnated with an oil‐in‐water cleaning emulsion and mint‐flavored oils. The cleaning emulsion combined with the mechanical action of the user wiping the denture was designed to freshen the denture and remove microbial biofilm and food debris. The formulation contains ingredients with an established use in oral health care products and/or that have generally recognized as having safe/food additive status and/or are listed in the US Food and Drug Administration inactive ingredient guide. The intended purpose of this experimental wipe is to allow denture wearers an opportunity to discretely and safely clean/refresh their dentures when away from home. While the ingredients incorporated in the wipe are considered benign, it is believed that there is no current published safety information demonstrating the tolerability of these ingredients in use as a wipe format. Several factors can impact oral and dermatological tolerability of oral care products. These factors include pH, the presence and levels of flavoring agents, detergents, and other excipients, along with the amount of product used. However, there appears to be no published evidence for the safety of any denture wipe in use, in particular whether any residue ingredients left on the dentures or on the hand of the user is generally well tolerated. The compatibility of the denture wipes with commonly used denture materials has been demonstrated (GSKCH, data on file).

The primary objective of the current study was to evaluate oral tolerability following 14 days’ use of an experimental, nonrinse, denture‐cleansing wipe to remove food from dentures. Secondary objectives were to determine the dermal tolerability of the denture‐cleansing wipe and its acceptability to users. The study also included an exploratory objective of developing a method to evaluate the effectiveness of the denture‐cleansing wipe in removing debris from denture surfaces.

## Materials and methods

This was a two‐part study conducted in healthy participants at a single center (Salus Research, Fort Wayne, IN). The study protocol was approved by an institutional review board (IRB; US IRB Miami, FL 33143; IRB number: USIRB2015SRI/18) and was registered with Clinicaltrials.gov: NCT03478644. The study was conducted in accordance with the Declaration of Helsinki and good clinical practice guidelines. All participants provided written informed consent to participate in the study before undergoing any study procedures. The study protocol was originally designed for the evaluation of a product with a slightly different formulation and was amended to allow investigation of the denture wipe described here. The protocol amendments were made before the start of the study; therefore, study flow and outcomes were unaffected.

### Study population

Study participants were recruited from a panel of individuals who wore either full or partial dentures, established previously from the local community. All dentures were required to be in good condition, defined as providing evidence of adequate vertical dimension, freeway space, horizontal occlusal relationships, and border extension; acceptable contour and finish; and acceptable porosity, tissue surfaces, polished surfaces, color, and thickness. For Part 2 of the study only, participants were required to have a full maxillary denture (with a full, partial, or no mandibular denture). All eligible participants were healthy, aged 18 to 80 years, were either fully or partially edentulous, and had a Fitzpatrick skin phototype classification of I (white, very fair with freckles) to IV (beige with a brown tint).^13^ Participants were excluded if they had a Fitzpatrick skin type of V or VI or had visible skin marks or conditions that would interfere with the evaluation of potential skin reactions. Key exclusion criteria included pregnancy or breastfeeding; known or suspected intolerance or hypersensitivity to the study materials; known allergy to any nuts or peanuts; history of an allergic reaction or feeling of discomfort to topically used products; use of denture adhesives; and the presence of oral soft tissue (OST) findings including stomatitis, open sores, lesions, redness, or swelling.

### Study procedures

The study comprised a screening/baseline visit and a 2‐week treatment period with clinic visits after 7 days (±3 days) and 14 days (±3 days) of study treatment use. At the screening visit, participant medical history and details of current/concomitant medications were recorded. An OST examination was conducted by a dental examiner, and dermatological assessment of both sides of the lead hand was performed by a dermatologist. The results of these assessments served as the baseline data.

For Part 1 of the study, all eligible participants were randomized (1:1) to 1 of 2 parallel treatment groups to use either the experimental denture wipes or a tap‐water rinse to clean their dentures. Participants were stratified by denture type: full or partial (Table 1). Randomization numbers within each stratum were assigned according to a computer‐generated randomization schedule supplied by the Biostatistics Department of GSKCH in ascending numerical order according to the sequence in which participants successfully met the inclusion/exclusion criteria at the baseline visit. Block randomization was used with a block size of 4. Both the dental examiner and dermatologist were blinded to the study treatment. Participants were instructed to use the denture wipe or tap‐water rinse to clean their dentures, external to their mouth, up to 4 times daily (once after completion of each of their 3 main meals and once at any other time) for 14 days.

**Table 1:** Summary of baseline characteristics (safety population)

**Characteristic**	**Denture wipe (n = 76)**	**Water rinse (n = 76)**
**Sex, n (%)**		
Male	19 (25.0)	28 (36.8)
Female	57 (75.0)	48 (63.2)
**Age, years**		
Mean (SD)	61.0 (12.70)	61.0 (12.62)
Median (range)	62.5 (22.0‐79.0)	63.0 (18.0‐80.0)
**Race, n (%)**		
White	64 (84.2)	67 (88.2)
Black or African American	10 (13.2)	8 (10.5)
Other	2 (2.6)	1 (1.3)
**Denture type, n (%)**		
Full^a^	39 (51.3)	38 (50.0)
Partial^b^	37 (48.7)	38 (50.0)

a Participants wearing full maxillary dentures, irrespective of type of mandibular dentition (full or partial dentures or natural) and participants with full mandibular dentures and natural maxillary dentition.

b Participants wearing partial maxillary dentures, irrespective of type of mandibular dentition (full or partial dentures or natural) and participants with partial mandibular dentures and natural maxillary dentition.

SD = standard deviation.

Participants randomized to the denture‐wipe group received a labeled pack (1 pack per denture) containing sufficient denture wipes for the duration of the treatment period. Participants were instructed to use 1 wipe per denture each time (i.e., participants with both a maxillary and mandibular denture used 2 wipes). The first use of the study treatment was undertaken and supervised at the study site. Participants were provided with a diary card to record all the wipes/water rinses used within the treatment period and an acceptability questionnaire to be completed at home immediately before and after their first home use of the study treatment.

To maintain denture hygiene, Polident® Overnight/Whitening denture‐cleansing tablets (GSKCH, Moon Township, PA) were provided to all participants for use overnight for the duration of the study. The use of denture cleansers other than those provided or denture fixatives was not permitted for the study duration. Changes to usual dietary habits, cosmetic use of hormonal therapy, exposure to excessive sunlight, and use of artificial tanning beds, nonsteroidal anti‐inflammatory drugs (continuous), corticosteroids, antihistamines, immunosuppressants, and vitamin A (and derivatives) were also not permitted during the study. The experimental denture wipe was not designed to adequately remove denture adhesive and/or to enable re‐application of denture adhesives; hence, participants were asked to refrain from adhesive use for the duration of the study.

Participants returned to the study site on days 7 and 14 for a repeat OST examination and dermatological assessment of the skin on their dominant hand and fingers. Participants were instructed not to rinse or wipe their dentures during the 2 hours before the post‐baseline study visits. Any adverse events (AEs) and medical‐device incidents were also recorded. Participants were requested to return their completed acceptability questionnaire to the study site on day 7.

### Dermal assessment

Skin irritation (SI) was determined by a dermatologist by examining the front and back of the participant's dominant hand (i.e., the hand used to hold the wipe, including fingers) and scored using a 6‐point scale (0 = no apparent cutaneous involvement; 0.5 = equivocal reaction; 1 = slight erythema with or without edema; 2 = moderate erythema with edema, with or without papules; 3 = severe erythema with edema, with or without papules; 4 = severe erythema with vesicles or blisters). Any site with a post‐baseline SI score ≥2 was to be categorized as an AE, visually assessed, and followed up until it resolved, or the participant was lost to follow‐up.

### Acceptability

Acceptability of the denture wipe and water rinse was evaluated using participant responses. Before and after the first time they cleaned their dentures at home according to their allocated procedure, participants were asked to rate: (i) How fresh does your denture feel? (ii) How clean does your denture feel (running the tongue over the denture teeth to judge)? (iii) How clean does your denture/smile look? (iv) How satisfied are you with the amount of debris removed after cleaning? Participants rated their responses as ‘not at all,’ ‘slightly,’ ‘moderately,’ ‘very,’ or ‘extremely.’

### Food (peanut particle) removal testing

For Part 2 of the study, after screening, enrolled participants who had full maxillary dentures were assessed for food (peanut particle) migration under their maxillary denture. Study site staff cleaned the dentures (Polident® 3 Minute denture‐cleansing tablet; GSKCH, Moon Township, PA), after which participants reseated their dentures then were instructed to consume a 30 to 32 g portion of nonsalted peanuts divided into portions of approximately 4 whole peanuts. Each portion was chewed for approximately 20 seconds, after which the participants were instructed to swallow when they felt comfortable. They then rinsed their mouth with tap water for 5 seconds before removing their maxillary denture. The extent of peanut‐particle migration under each denture was visually assessed by the study investigator and scored using a 4‐point scale (where 0 = none [no peanut migration under the denture]; 1 = minimal [slight migration under the denture]; 2 = moderate [peanut migration over the internal walls of the denture]; 3 = extensive [peanut migration on the crest of the denture]). Participants with a peanut‐particle migration score of >0 on the maxillary denture were eligible for inclusion in the food‐removal assessment part of the study.

The first 32 participants who met the criteria for adequate food migration after consuming the standardized portion of peanuts were then instructed to clean their maxillary denture using one of the experimental denture wipes. The denture and wipe were collected and placed in separate labeled beakers. Water (150 mL) was added to each beaker before sonication for approximately 15 minutes to loosen and wash out any peanut particles remaining on the denture or denture wipe into the water in their respective beakers. The denture and wipe were carefully taken out of the beakers so as not to remove any peanut particles from the beakers. The denture was cleaned with a denture cleanser and denture brush and returned to the participant; the wipe was discarded.

The solutions in the beakers were sieved, and the sieved residue was rinsed repeatedly with hot water to remove any saliva. After air‐drying overnight, the collected peanut particles and residue were dried on preweighed aluminum weighing pans in an oven at 40°C for 5 hours. The aluminum pans were then weighed to determine the weight of peanut particles (in mg) retrieved by the denture wipe and the residual weight of peanuts on the denture.

For a subset of participants (2 participants per peanut‐particle migration‐scale score), dentures were photographed after peanut consumption both before and after use of the denture wipe. The photographs were used by study site staff to ensure consistent evaluation throughout this part of the study.

### Safety

AEs and SI findings reported after administering the study treatments were used in the safety assessment. The primary safety endpoint was the proportion of participants experiencing or reporting oral treatment‐emergent AEs (TEAEs) in each treatment group on or before day 14. The proportion of participants experiencing or reporting any TEAE in each treatment group on or before day 7 was also determined.

OST examinations were performed by a dental examiner using direct observation and palpation with retractable aids as appropriate. Any new or worsening abnormality from the screening assessment was recorded as an AE.

### Statistical analyses

No formal sample‐size determination was performed. Approximately 160 eligible participants (approximately 80 full denture wearers and 80 partial denture wearers) were planned to be randomized to Part 1 of the study to ensure that at least 150 participants (approximately 75/treatment group) completed the study. Approximately 35 eligible participants were planned to enter the food‐removal testing part of the study (Part 2) to ensure that a minimum of 30 participants completed the food‐removal assessment.

The safety population included all participants who were randomized and received treatment at least once during the study. The modified intent‐to‐treat (mITT) population (Part 2 only) included all eligible participants with full maxillary dentures who used the denture wipe in the food‐removal testing part of the study and provided data for the food‐removal assessment. No missing data imputation method was used.

The numbers, percentages, and 95% (Clopper–Pearson) confidence intervals (CIs) were calculated for the proportion of participants in each treatment group (safety population) experiencing a TEAE, oral TEAE, or treatment‐related TEAE by days 7 and 14. No formal statistical comparisons were made between the treatment groups. Analysis of SI scores was performed on the safety population; the numbers and percentages of participants with each SI score were recorded, as was proportion of participants experiencing SI scores of ≥2 in each treatment group after 7 and 14 days’ use. Responses to the acceptability questionnaire were tabulated and listed as the numbers and percentages of participants.

Descriptive results were determined for the weight of retrieved peanut particles and the weight of peanut particles remaining on the dentures in the mITT population. The percentage of food removed was calculated as:

100 × (weight of peanut particles retrieved/[weight of peanut particles retrieved + weight of peanut particles remaining on the denture]).

## Results

**Figure 1:**
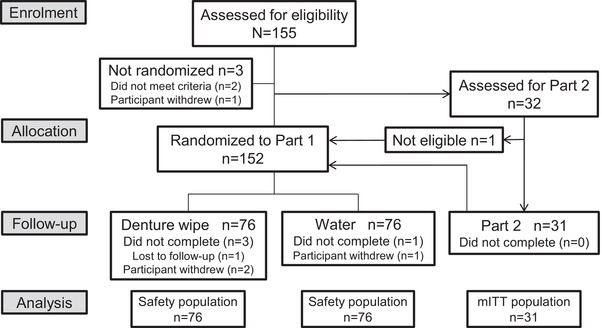
Participant disposition. mITT = modified intent‐to‐treat.

The study was conducted between October 10, 2016 and October 31, 2016. In Part 1, 152 participants were randomized to the study (including those in Part 2) (denture wipe, n = 76; water rinse, n = 76) and comprised the safety population (Fig 1). Participants had a mean age of 61.0 years (range 18‐80 years), and the majority were women (69.1%). The safety population was well balanced by denture type (Table 1). A total of 2033 denture wipes and 1969 water rinses were used by participants during the study. For Part 2 (whose participants were a subset of those in Part 1), 32 participants were assessed for eligibility, and 31 completed this part of the study (mITT population).

### Safety

The proportions of participants experiencing a TEAE by days 7 and 14 were, respectively, 0.013% (95% CI: 0.00, 0.07) and 0.039% (95% CI: 0.01, 0.11) in the denture‐wipe group and 0.000% (95% CI: 0.00, 0.05) and 0.013% (95% CI: 0.00, 0.07) in the water‐rinse group. Four TEAEs were reported in 4 participants (2.6%; Table 2). All were oral TEAEs and included lip injury by day 7 (1 participant) and mouth injury by day 14 (2 participants) in the denture‐wipe group and a coated tongue by day 14 in the water‐rinse group (1 participant).

**Table 2:** Treatment‐emergent adverse events (TEAEs) (safety population)

	**Denture wipe (n = 76)**				**Water rinse (n = 76)**			
	By day 7		By day 14^a^		By day 7		By day 14^a^	
**TEAE**	n (%)	nAE	n (%)	nAE	n (%)	nAE	n (%)	nAE
**At least one TEAE**	1 (1.3)	1	3 (3.9)	3	0	0	1 (1.3)	1
Non‐oral TEAE	0	0	0	0	0	0	0	0
**Oral TEAE**	1 (1.3)	1	3 (3.9)	3	0	0	1 (1.3)	1
Lip injury	1 (1.3)	1	1 (1.3)	1	0	0	0	0
Mouth injury	0	0	2 (2.6)	2	0	0	0	0
Coated tongue	0	0	0	0	0	0	1 (1.3)	1

a Cumulative data so that day 14 includes TEAEs that were also recorded at day 7.

n (%) = number (percent) of participants; nAE = number of adverse events.

All TEAEs were mild in intensity except coated tongue, which was moderate in intensity. The TEAE of lip injury (mild erythema of the upper lip) was considered treatment‐related. The mouth injuries were both traumas due to bites, thus not considered treatment‐related. The TEAE of coated tongue was the only TEAE deemed not to have resolved by the end of the study (participant lost to follow‐up). There were no non‐oral TEAEs and no serious TEAEs. Withdrawal (2 in the denture‐wipe group, 1 in the water‐rinse group) was due to the participants being unable to commit to the study schedule; no participant withdrew from the study due to a TEAE. There were no medical‐device incidents recorded.

### Dermal assessment

All participants in the denture‐wipe group had an SI score of 0 at baseline (Table 3); this remained unchanged throughout the study. 4 participants in the water‐rinse group had baseline scores of 1. After 7 and 14 days of use, 3 participants scored 1 on each day, with the remaining participants scoring 0. Only one participant, who was randomized to the water‐rinse treatment, experienced a deterioration in SI score (from 0 at day 7 to 1 at day 14) during the study.

**Table 3:** Skin irritation scores (safety population)

	**Denture wipe (n = 76)**			**Water rinse (n = 76)**		
**Score**	Day 0	Day 7	Day 14	Day 0	Day 7	Day 14
0	76 (100)	74 (100)	73 (100)	72 (94.7)	73 (96.1)	72 (96.0)
0.5	0	0	0	0	0	0
1	0	0	0	4 (5.3)	3 (3.9)	3 (4.0)
2	0	0	0	0	0	0
3	0	0	0	0	0	0
4	0	0	0	0	0	0
Missing^a^	0	2	3	0	0	1

All data are n (%).

a Missing values are not included in denominators for percentage calculations.

### Acceptability questionnaire

**Figure 2:**
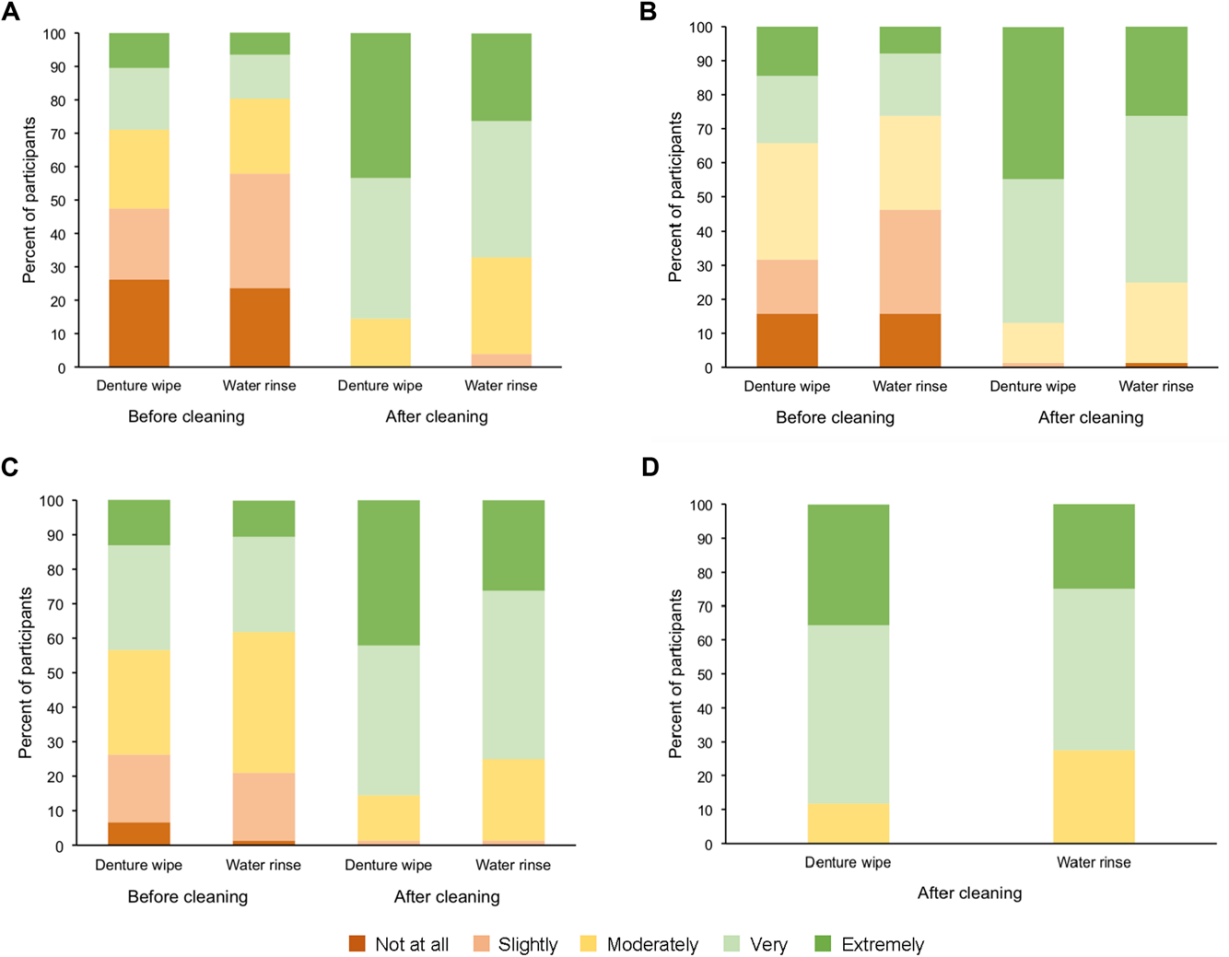
Participant responses to the acceptability questionnaire: (A) How fresh does your denture feel? (B) How clean does your denture feel? (C) How clean does your denture/smile look (in the mirror)? (D) How satisfied are you with the amount of debris you removed?.

Participant responses to the acceptability questionnaire are summarized in Figure 2 and Table 4. For the question ‘How fresh does your denture feel?,’ after eating and before cleaning, 28.9% in the denture‐wipe group and 19.8% in the water group rated their dentures as feeling ‘extremely’ or ‘very’ fresh. Post‐cleaning percentages were 85.5% for the denture‐wipe group, 67.1% for the water‐rinse group. For the question ‘How clean does your denture feel?’ respective percentages of those answering ‘very’/’extremely’ clean for the denture‐wipe/water‐rinse groups were 34.2%/26.3% pre‐cleaning and 86.8%/75.0% post‐cleaning. For the question ‘How clean does your denture/smile look (in the mirror), respective pre‐ and post‐cleaning ‘very’/’extremely’ clean percentages for the denture‐wipe/water‐rinse groups were 43.5%/38.1% and 85.5%/75.0%.

**Table 4:** Participant responses to the acceptability questionnaire

	**After eating and before cleaning denture**		**After removing and cleaning denture**	
**Question**	Denture wipe (n = 76)	Water rinse (n = 76)	Denture wipe (n = 76)	Water rinse (n = 76)
**How fresh does your denture feel?**				
0: Not at all	20 (26.3)	18 (23.7)	0	0
1: Slightly	16 (21.1)	26 (34.2)	0	3 (3.9)
2: Moderately	18 (23.7)	17 (22.4)	11 (14.5)	22 (28.9)
3: Very	14 (18.4)	10 (13.2)	32 (42.1)	31 (40.8)
4: Extremely	8 (10.5)	5 (6.6)	33 (43.4)	20 (26.3)
**How clean does your denture feel?**				
0: Not at all	12 (15.8)	12 (15.8)	0	1 (1.3)
1: Slightly	12 (15.8)	23 (30.3)	1 (1.3)	0
2: Moderately	26 (34.2)	21 (27.6)	9 (11.8)	18 (23.7)
3: Very	15 (19.7)	14 (18.4)	32 (42.1)	37 (48.7)
4: Extremely	11 (14.5)	6 (7.9)	34 (44.7)	20 (26.3)
**How clean does your denture/smile look (in the mirror)?**				
0: Not at all	5 (6.6)	1 (1.3)	0	0
1: Slightly	15 (19.7)	15 (19.7)	1 (1.3)	1 (1.3)
2: Moderately	23 (30.3)	31 (40.8)	10 (13.2)	18 (23.7)
3: Very	23 (30.3)	21 (27.6)	33 (43.4)	37 (48.7)
4: Extremely	10 (13.2)	8 (10.5)	32 (42.1)	20 (26.3)
**How satisfied are you with the amount of debris you removed?**				
0: Not at all	–	–	0	0
1: Slightly	–	–	0	0
2: Moderately	–	–	9 (11.8)	21 (27.6)
3: Very	–	–	40 (52.6)	36 (47.4)
4: Extremely	–	–	27 (35.5)	19 (25.0)

All data are n (%).

### Food removal

**Figure 3:**
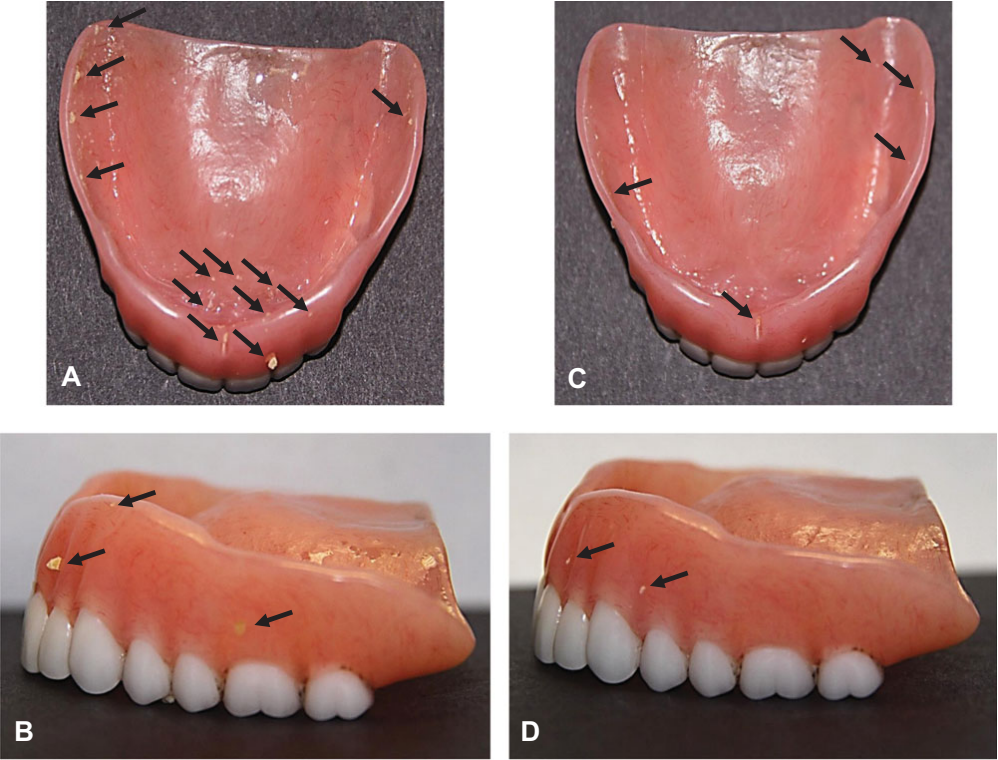
Typical photograph of a denture before (A and B) and after (C and D) use of the experimental denture wipe (arrows indicate location of peanut particles). Figure constructed using Microsoft® PowerPoint® 2016 MSO (Microsoft Corp., Redmond, USA); overall sharpness, color balance, and brightness of each photo have been adjusted to allow for easier visualization of residue.

When asked ‘How satisfied are you with the amount of debris you removed?,’ a higher proportion of participants in the denture‐wipe group were extremely satisfied (35.5%) or very satisfied (52.6%) compared with participants in the water‐rinse group (25.0% and 47.4%, respectively) (Fig 2, Table 4). Figure 3 shows a typical photograph of the dentures before and after using the denture wipe. The mean weight of peanut‐particle residue removed after a single use of the denture wipe was 2.57 mg (standard error = 0.71 mg). The mean weight of peanut‐particle residue remaining on the denture was 0.71 mg (standard error = 0.31 mg). This equated to removal by the denture wipe of 78.4% of the peanut‐particle debris that had adhered to the denture during mastication.

## Discussion

There is an unmet need for added convenience for denture cleansing, enabling denture wearers to remove irritating food particles, freshen the denture, and reduce the bio‐burden on a more frequent basis and in a more convenient manner. Conventional cleansing methods do not meet all of these requirements. Soaking and brushing with a tablet denture cleanser or brushing with toothpaste requires equipment and time; rinsing under running water can be effective at removing debris but does not offer the convenience or freshening provided by the experimental denture wipes under investigation.

This study was primarily conducted to assess the tolerability of the denture wipes before ensuring their safety for users, and to confirm the hypothesized low risk of dermal and oral irritation. The ingredients used in this formulation have an acceptable safety profile at the concentrations selected and an acceptable margin of safety between anticipated exposure to the ingredients and an exposure level that might result in adverse effects. The ingredients also have established use in oral health care products.

Here, the oral and dermal tolerability of the experimental denture wipe was demonstrated after 14 days of use. The study is therefore in agreement with the expected tolerance. No scores breached the preset criteria to be considered an AE in either treatment group. The population selected for the study and the target consumers for the wipes are anticipated to have greater dermal fragility than average in the population because they normally comprise an older age group. It was interesting to note that more participants showed greater skin‐irritation scores with the water rinse than the denture wipes. Given the additional wetting of the hands during rinsing for this group, these results may not be surprising.

As the wipes are designed such that dentures do not require rinsing following use, the flavor in the formulation is intended to leave the denture wearer feeling that their denture is freshened and the wearer is less self‐conscious of denture malodor, another main concern.^5^ Following use, more participants in the denture‐wipe group than the water‐rinse group rated their dentures as feeling very/extremely fresh and feeling/looking very/extremely clean. Acceptance of the wipe could improve use and compliance and the benefits linked to this regarding the freshening and removal of food and microorganisms.

The formulation of the denture wipes was designed to lift and remove food debris and denture plaque/biofilm from dentures. Food trapped between the denture and the oral tissue can be uncomfortable for a denture wearer and may leave an area of irritation. Denture wearers will typically attempt to remove the trapped food/debris by rinsing with water.^12^ If the person is not in a home environment this can be difficult; for example, the stigma of wearing a denture may make the person feel uncomfortable taking their denture out and rinsing it in a public washroom. The denture wipes enable the denture wearer to remove food debris in a more private setting.

The acceptability of the treatment, as evaluated using a questionnaire, indicated that a higher proportion of participants who used the denture wipe were very/extremely satisfied with removal of debris from their denture compared with participants who used the water rinse. The exploratory method used to evaluate the effectiveness of the denture wipe in removing food debris demonstrated that the denture wipe adequately removed more than three‐quarters of the particles from dentures after eating. As such, this methodology is suitable for further investigations that examine the action and effectiveness of the denture wipe.

When used as an adjunct to conventional cleaning (e.g., with a tablet cleanser), the wipes, with their physical mode of action, have the potential to reduce the denture bio‐burden at more frequent intervals, which, in turn, may improve the efficacy of the chosen primary cleaning technique. The ability for the experimental denture wipe to remove bacteria has been established in in vitro studies (GSKCH data on file); however, this does not imply the wipe could replace more conventional and thorough denture‐cleansing methods with proven efficacy in reducing bio‐burden and killing microorganisms associated with denture malodor and stomatitis.

## Conclusions

1. Overall, the experimental denture wipes were well received by the study participants, with no tolerability issues observed during the study and satisfactory removal of food particles.
2. Acceptability with regard to the dentures being fresh, clean feeling, and clean looking was high.
3. Use of the wipe may lead to improved quality of life for the denture wearer, potentially reducing the physical pain caused by food irritations and thereby possibly increasing their social confidence.
4. Data from this initial study aimed at investigating the tolerance of the wipes encourage further studies into the impact on overall bio‐burden during the day, the impact on the efficacy of the main daily cleansing method, the impact on the quality of life of the denture wearer, and the effectiveness of the wipe at removing food and oral debris.
